# Molecular Markers Help with Breeding for Agronomic Traits of Spring Wheat in Kazakhstan and Siberia

**DOI:** 10.3390/genes15010086

**Published:** 2024-01-10

**Authors:** Alexey Morgounov, Adylkhan Babkenov, Cécile Ben, Vladimir Chudinov, Yuriy Dolinny, Susanne Dreisigacker, Elena Fedorenko, Laurent Gentzbittel, Awais Rasheed, Timur Savin, Sergey Shepelev, Rauan Zhapayev, Vladimir Shamanin

**Affiliations:** 1 Faculty of Agronomy, Kazakh Agrotechnical University Named after S. Seyfullin, Astana 010000, Kazakhstan; 2 A.I. Barayev Research and Production Centre for Grain Farming, Shortandy 021601, Kazakhstan; babkenov64@mail.ru (A.B.); ura_dolin@mail.ru (Y.D.); savintimur_83@mail.ru (T.S.); 3 Project Center for Agrotechnologies, Skolkovo Institute for Science and Technology, 121205 Moscow, Russia; c.ben@skoltech.ru (C.B.); l.gentzbittel@skoltech.ru (L.G.); 4 Karabalyk Agricultural Experimental Station, Kostanay 110000, Kazakhstan; ch.den@mail.ru; 5 International Maize and Wheat Improvement Center, P.O. Box 041, Texcoco 100, Mexico; s.dreisigacker@cgiar.org; 6 North Kazakhstan Agricultural Experimental Station, Shagalaly 150311, Kazakhstan; efedorenko2015@mail.ru; 7 Department of Plant Sciences, Quaid-i-Azam University, Islamabad 45320, Pakistan; arasheed@qau.edu.pk; 8 International Maize and Wheat Improvement Center, Beijing 100081, China; 9 Faculty of Agrotechnology, Omsk State Agrarian University, 644008 Omsk, Russia; ss.shepelev@omgau.org (S.S.); vp.shamanin@omgau.org (V.S.); 10 Kazakh Scientific Research Institute of Agriculture and Plant Growing, Almaty 040909, Kazakhstan; r.zhapayev@mail.ru

**Keywords:** cereals, DNA markers, grain yield, genotypes-environment interaction

## Abstract

The Kazakhstan-Siberia Network for Spring Wheat Improvement (KASIB) was established in 2000, forming a collaboration between breeding and research programs through biannual yield trials. A core set of 142 genotypes from 15 breeding programs was selected, genotyped for 81 DNA functional markers and phenotyped for 10 agronomic traits at three sites in Kazakhstan (Karabalyk, Shortandy and Shagalaly) and one site in Russia (Omsk) in 2020–2022. The study aim was to identify markers demonstrating significant effects on agronomic traits. The average grain yield of individual trials varied from 118 to 569 g/m^2^. Grain yield was positively associated with the number of days to heading, plant height, number of grains per spike and 1000-kernel weight. Eight DNA markers demonstrated significant effects. The spring-type allele of the *Vrn-A1* gene accelerated heading by two days (5.6%) and was present in 80% of the germplasm. The winter allele of the *Vrn-A1* gene significantly increased grain yield by 2.7%. The late allele of the earliness marker per se, *TaMOT1-D1,* delayed development by 1.9% and increased yield by 4.5%. Translocation of 1B.1R was present in 21.8% of the material, which resulted in a 6.2% yield advantage compared to 1B.1B germplasm and a reduction in stem rust severity from 27.6 to 6.6%. The favorable allele of *TaGS-D1* increased both kernel weight and yield by 2–3%. Four markers identified in ICARDA germplasm, *ISBW2-GY* (Kukri_c3243_1065, 3B), *ISBW3-BM* (TA004946-0577, 1B), *ISBW10-SM2* (BS00076246_51, 5A), *ISBW11-GY* (wsnp_Ex_c12812_20324622, 4A), showed an improved yield in this study of 3–4%. The study recommends simultaneous validation and use of selected markers in KASIB’s network.

## 1. Introduction

Spring bread wheat is a major crop grown on over 16–17 M ha in North Kazakhstan and Western Siberia of Russia. This is a short-season crop sown in May and harvested in August–September. The production system includes other cereals (spring durum wheat, barley and oats), oil crops (sunflower, rapeseed, flax) and legumes (dry peas, lentil and chickpea). The spring wheat is produced entirely under rainfed conditions, and moisture stress is the key abiotic challenge limiting grain yield. Diseases (leaf and stem rusts, *Septoria* sp. and tan spot) and numerous insect pests occur regularly and limit production. The average grain yield in the region varies from 1.2 to 1.8 t/ha depending on the year [1]. Utilization of low-input production technologies and conventional tall cultivars is one of the reasons for the low yield. North Kazakhstan and Western Siberia are important for regional and global food security since most of the grain produced here is exported (www.fao.org/faostat, accessed on 1 September 2023). Therefore, raising the productivity and stability of wheat farming is an important task.

The wheat breeding framework in the region comprises primarily public breeding programs located at agricultural research stations, institutes and centers across the main agro-ecological regions [2]. The breeding system is based on crosses with subsequent selection using a modified pedigree method and yield testing at the advanced breeding stages. The development of new varieties takes 10–12 years, followed by 2–3 years of official registration trials and another 3–5 years for seed production, making it almost 20 years from the cross to the farmer’s field [2]. Very few programs in the region utilize modern genomic and phenomic tools. Multilocational trials at advanced breeding stages are also very limited. As a result, many released cultivars are susceptible to diseases, are tall and lodge in favorable environments, are not responsive to inputs and have limited adaptations. Several locally developed cultivars have been officially released but have not been grown at a large scale. In the meantime, there is competition from foreign, primarily European, cultivars with short stature, which are favored by farmers due to their being more technologically suitable and responding well to inputs. There is an obvious need for modernization of the breeding programs, including the utilization of genomic and phenomic tools.

DNA molecular markers have emerged as an important instrument for the selection of genotypes with desirable traits [3]. This constantly evolving technology offers high selection efficiency for various wheat traits, including resistance to biotic and abiotic stresses, adaptation, agronomic performance and grain quality. Different types of wheat molecular markers and their use in breeding have been summarized recently by Song et al. [4] and Sun et al. [5]. The research on the genetics and identification of molecular markers of spring wheat in North Kazakhstan and Western Siberia focused on grain elemental composition, leaf rust [6,7] and agronomic traits [8,9]. However, the outcomes of these studies have not been implemented in practical wheat breeding.

The Kazakhstan-Siberia Network for Spring Wheat Improvement (KASIB) was established in 2000, uniting more than 20 breeding and research programs. The network objective has been to exchange the germplasm and conduct cooperative multilocational testing to characterize the advanced breeding lines and new cultivars. Each participating institution submits 2–3 entries for a cooperative trial that lasts for 2 years. The resulting data are integrated and distributed among the network members. By 2018, more than 300 genotypes had been exchanged and tested in KASIB yield trials. KASIB’s core set, with 142 genotypes, was selected from this material. The set was genotyped for over 80 functional markers and phenotyped for three years at four sites. The study objective was to evaluate the effects of molecular markers on adaptation and productivity traits for utilization in practical breeding.

## 2. Materials and Methods

### 2.1. Material

**Figure 1:**
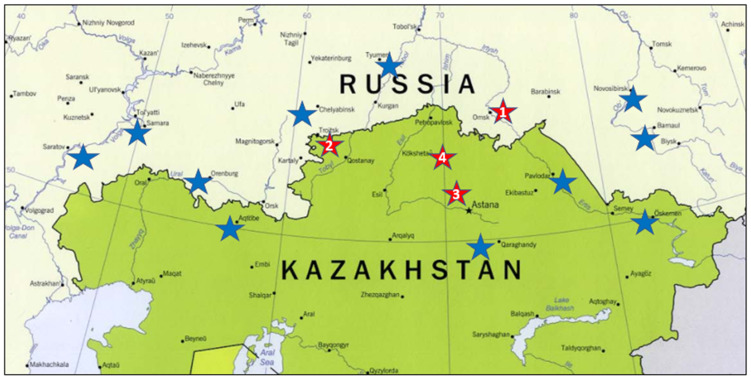
Location of KASIB trials used in the study: 1. Omsk State Agrarian University, Omsk (55.026333 N, 73.310254 E); 2. Karabalyk Agricultural Experimental Station, Karabalyk, Kostanay reg. (53.845009 N, 62.122684 E); 3. A.I. Barayev Research and Production Centre for Grain Farming, Shortandy, Akmola reg. (51.636491 N, 71.021044 E); 4. North Kazakhstan Agricultural Experimental Station, Shagalaly, North Kazakhstan reg. (54.163557 N, 69.521307 E). Blue stars indicate KASIB sites not included in the study.

A KASIB core set comprising 142 genotypes (Table S1) was selected from 325 KASIB accessions exchanged between cooperators from 2001 to 2018. The selection was made based on: (a) DNA diversity as represented by 63 random KASP-SNP markers, selection was made from different clusters developed by COREHUNTER software, v3; (b) geographic representation to include germplasm from all KASIB cooperators; and (c) agronomic performance including the five highest yielding genotypes from each KASIB trial. The germplasm represented eight breeding programs from Kazakhstan totaling 59 entries and nine programs from Russia with 83 entries. Geographic origin of the germplasm is presented in Table S1 and Figure 1. KASIB cooperators are located in diverse environments, varying in soil, climate and cropping systems. The distance from the westernmost site, Saratov to the easternmost, Barnaul is over 3000 km, while northernmost location, Tyumen is over 2000 km from the southernmost, Almaty. Most of the germplasm tested were advanced lines, while cultivars accounted for around 35%. The seeds were initially multiplied at Karabalyk Agricultural Experimental Station (AES) in 2018, distributed to three other sites and multiplied there in 2019, allowing replicated trials starting from 2020 with each site using their own seeds.

### 2.2. Yield Trials

The yield trails were conducted at four sites (Figure 1): Omsk State Agrarian University, named after P. Stolypin, (3 m^2^ plot; 2020 and 2022—4 reps; 2021—2 reps), Karabalyk AES (3 m^2^ plot; 2020 and 2022—2 reps; 2021—3 reps), A.I. Barayev Research and Production Centre for Grain Farming (3 m^2^ plot; 2020 and 2022—1 rep; 2021—2 reps), North Kazakhstan AES (5 m^2^ plot; 2021 and 2022—2 reps). The number of replications varied depending on individual program choices in a particular year to accommodate labor-intensive phenotyping. The experiments were laid out using a randomized complete block design. The following agronomic traits were recorded: number of days from emergence to heading and from heading to maturity, plant height (plot average), number of total and productive tillers per plant, spike length, number of grains per spike, 1000-kernel weight (TKW), grain weight per spike following the methodology described by Pask et al. [10] using 5 random plants per replication. Stem rust severity was evaluated as average per plot [10]. The trials were harvested by combine. ANOVAs for unbalanced design and associated analyses were conducted using R 4.3.0 using the sommer package. Best Linear Unbiased Predictions (BLUP) and Best Linear Unbiased Estimates (BLUE) for six target traits were calculated to account for unbalanced design as described by Smith et al. [11].

The preceding field was black fallow at all sites. Sowing took place at optimal dates between 15 and 25 of May. The seed rate varied from 300 to 400 seeds per m^2^ depending on location. Weeds were controlled with common herbicides. No disease or insect protection took place. The fertilizers were not applied following common production practices after fallow.

The soils across the study locations represented various types of chernozem: ordinary (Karabalyk), leached (Shortandy and Shagalaly) and meadow (Omsk) with pH within 6.5–7.5, average availability of phosphorus and high potassium. Nitrogen supply was above average. The weather conditions varied across the three years of study (Table 1), with 2020 being extremely dry and hot with the average temperature in July 1–2 °C higher than long-term values. Karabalyk site was exposed to moisture stress and heat in 2020 and 2022 as well. The growing conditions of 2022 were close to the long-term average in Omsk, Shortandy and Shagalaly. Among the diseases, only stem rust in Omsk in 2020 was observed, but it did not affect the yield performance. Overall, variable weather at four sites during three seasons allowed for a comprehensive evaluation of the germplasm.

### 2.3. Genotyping

DNA extraction was conducted from lyophilized tissue, followed by quantification, quality control and DNA purification as described in CIMMYT wheat molecular genetics: Laboratory Protocols and Applications to Wheat Breeding [12]. Kompetitive Allele Specific PCR (KASP) markers for 81 different loci were used (Table S3) with analysis performed by Biosearch Technologies (Teddington, Middlesex, UK). The primer sequences, amplification conditions and detailed genotyping procedures of some genes are described in [13], while for some genes this information was provided by respective research groups. The markers selection was based on a standard set of markers available at CIMMYT and ICARDA for wheat at the time of analysis in 2019. KASP markers for which one of the alleles was represented at a frequency higher than 80% were not considered for evaluation of marker–trait association. For several markers, especially disease and insect resistance, phenotyping was not conducted. Eventually, only 17 markers on 12 chromosomes were selected for evaluation in the study: development rate—5 loci, disease resistance—1 locus, plant height—1 locus, yield and yield components—10 loci. The marker–trait associations were identified by comparing the average values of BLUPs or original data with alternative marker alleles using the respective standard errors.

## 3. Results

### 3.1. Grain Yield and Agronomic Traits across the Study Sites and Years

**Figure 2:**
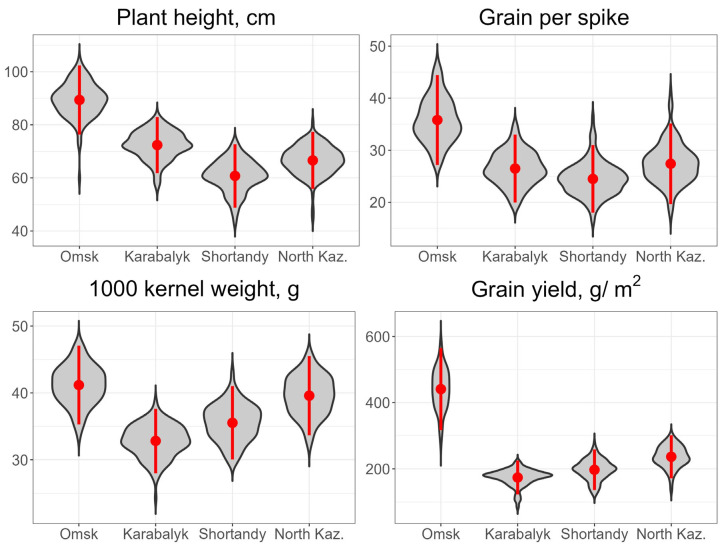
Best Linear Unbiased Estimates of agronomic traits at the four experimental sites in 2020–2022. Mean and confidence interval of the mean (95%) are indicated by a red point and red bar, respectively, within each violin plot.

The average values of grain yield and other agronomic traits for the four sites across the years are presented in Figure 2 and yearly data in Table S3. The Omsk environment was the most favorable for spring wheat compared to the three sites in Kazakhstan. The average grain yield in Omsk was 478 g/m^2,^ compared to 178–249 g/m^2^ at other sites. The plant height in Omsk was 88.3 cm for three years, or 17.4–45.2% taller than in other sites. Among yield components, spike fertility, reflected by number of grains per spike, was 35.6 compared to 24.1–26.8 at the sites in Kazakhstan. However, the Omsk trials were marginally superior to those of the North Kazakhstan station for 1000-kernel weight: 40.8 vs. 39.1 g. The lowest TKW was in Karabalyk—31.5 g. ANOVA results demonstrated highly significant effects of sites, years, genotypes and the interactions genotypes - sites, sites x years for all traits (height, spike length, number of grains per spike, grain weight per spike, TKW and grain yield) (Table S4). However, the interaction genotypes - years was only significant for TKW.

**Figure 3:**
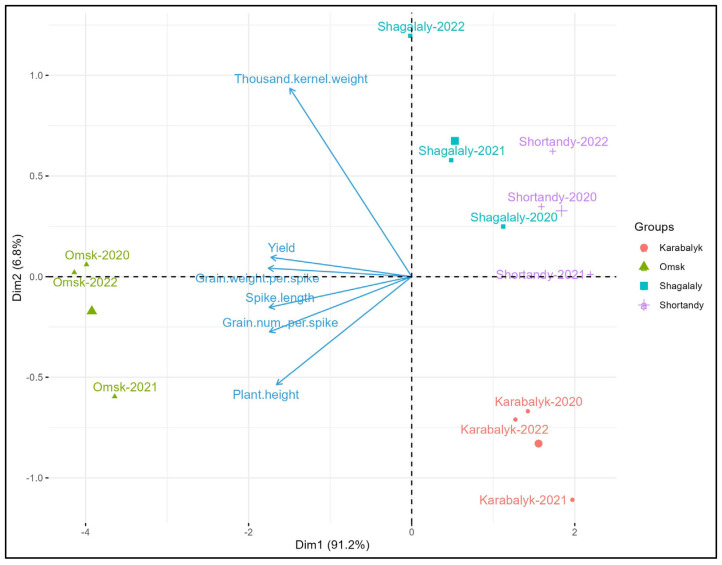
Biplot analysis of 12 trials using average yearly values of variety BLUEs.

Biplot analysis of 11 trials using average yearly values of 6 traits demonstrated the differences between the years and sites (Figure 3). Three seasons in Omsk were quite similar and formed one compact group. Similarly, the three seasons at Karabalyk are also grouped. Shortandy and Shagalaly define two other groups in the same quadrat of the biplot, suggesting similar performances of the spring wheat panel at these two locations. The expression of all traits was the highest in Omsk, as indicated by the length and direction of the arrows. The combinations of agronomic traits in Karabalyk in 2021 and Shagalaly in 2022 were quite unique, setting these sites apart from each other and from other trials. Overall, the 12 sites-years combinations were sufficiently diverse to allow the comprehensive evaluation of KASIB core material for adaptation and agronomic traits.

### 3.2. Association of Grain Yield with Adaptation Traits and Yield Components

Pearson coefficients of correlations were calculated between grain yield and yield component traits across all 142 genotypes for each site and year separately (Table 2) using BLUEs. Plant height had a significant positive correlation in all nine trials. Taller germplasm tended to provide consistently higher yields, especially in Shagalaly and Shortandy. The number of grains per spike had a higher association with the grain yield compared to TKW, considering individual locations. The respective coefficients of correlation were significant in 10 trials out of 11, compared to 6 for TKW. The grain weight per spike, as an integral trait, had as high of an association with grain yield as did the number of grains per spike. The correlations computed for average values for each year across sites are much higher compared to individual trials, and all five traits demonstrate high and positive association with grain yield. This means that all agronomic traits included in this study are important in defining spring wheat productivity in the target environments.

**Figure 4:**
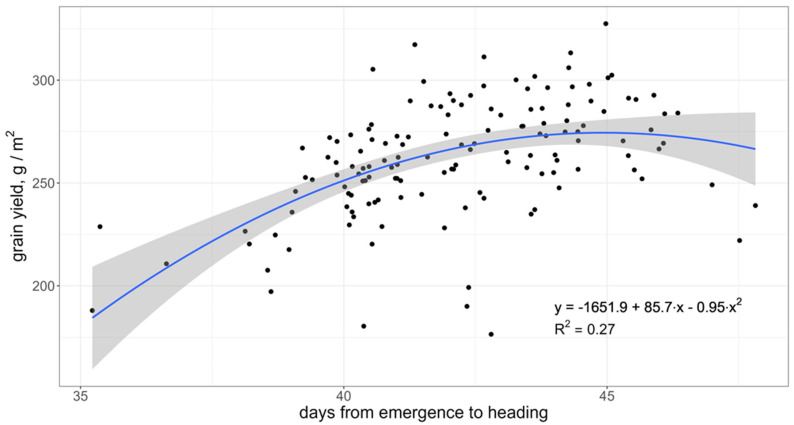
Relationship between the number of days to heading and grain yield computed from BLUE of the 142 accessions for the set of target environments. The grey area represents 95% confidence interval of the regression.

The rate of development or earliness measured by the number of days from emergence to heading is a key character for short-season wheat like in North Kazakhstan and Siberia. There is a clear positive association between the number of days to heading (within a range of 35–45 days) and grain yield (Figure 4). However, the later-heading and -maturing genotypes demonstrated lower yield, perhaps due to exposure to unfavorable conditions during maturity. The number of days from heading to maturity had a significant negative correlation with the number of days to heading (0.59) and an insignificant one with the grain yield.

The adaptation traits and yield components studied in this experiment had various degrees of association with the grain yield, which also depended on the site and year. This provides an optimal basis for evaluation of the effects of molecular markers on these traits.

### 3.3. Effects of Molecular Markers on Agronomic Traits

The *Rht8* allele of the Ribonuclease H-Like 1 gene [14] was present in most germplasm (78%) but did not reduce the height compared to the group of germplasm possessing the recessive allele (Table 3). Five markers linked to the genes controlling the rate of development, i.e., the number of days from emergence to heading, were assessed in the KASIB core set. The spring allele of the *Vrn-A1* gene accelerated heading by two days (5.6%), with a significant difference in average values across all trials and for each individual trial. This allele was present in 80% of the germplasm. The presence of the winter allele of the *Vrn-A1* gene significantly increased grain yield by 2.7%. The second marker with a significant effect on heading date was *TaMOT1-D1,* which accelerated development by 1.9% across all trials and was significant in five trials out of six. The presence of the allele which extends the growing period also significantly increased yield by 4.5%.

Two markers with effects on 1000-kernel weight and grain yield were evaluated: alleles at GS5-2334-SNP did not result in significant differences in either of the traits, while *TaGS-D1* significantly increased grain weight and yield by 2.1% and 3.2%, respectively. Marker *ISBW10-SM2-QTL,* with an effect on tillering capacity, proved its effect under KASIB conditions, significantly increasing the number of total and productive tillers per plant by 7.8 and 4.3%, respectively. However, it did not have a significant effect on yield. Two markers linked to genes affecting yield performance under drought conditions (*TaCwi-4A* and *Dreb1*) did not prove effective in the current study. They had no effect even in the trials with low yield caused by moisture stress.

For grain yield, the presence of the respective alleles of the markers *ISBW3-BM-QTL*, *ISBW11-GY-QTL* and *ISBW2-GY-QTL* increased the productivity by 3.5–4.5% across all sites and were also effective in most individual trials. Marker *ISBW11-GY-QTL* increased grain yield in Omsk in three years by 7.4%.

Translocation 1B.1R was present in 21.8% of the material studied. The groups of material possessing 1B.1R had a 6.2% yield advantage compared to the 1B.1B group across all 11 trials. However, its positive effect on yield in Omsk was 12.3% during three years, in North Kazakhstan—7.5%, in Shortandy—6.2%, while no advantage was found under Karabalyk conditions. The presence of 1B.1R translocation substantially reduced stem rust severity from 27.6 to 6.6%. Another marker of stem rust resistance, *IWAB8036,* did not significantly affect the pathogen severity.

### 3.4. Markers Distribution across the Breeding Programs

The allelic frequencies of eight effective markers described above were estimated in 322 cultivars and breeding lines originating from 15 breeding programs in Kazakhstan and Russia (Table 4). Allele *vrn-A1,* associated with winter growth habit, is considered favorable for productivity due to a positive association between the number of days to heading and grain yield (Figure 4). The frequency of this allele varied from zero in Aktobe, Chelyabinsk and Novosibirsk to 95% in the East Kazakhstan breeding program. Its presence in Kazakh germplasm was almost four times more frequent than in Russian material. The frequency of 1B.1R translocation was below 5% at six breeding programs in Kazakhstan, while it exceeded 20% at five programs in Russia. Its frequency in the germplasm from Kurgan Seeds company and Omsk Agricultural Research Center was 57.1%. Overall, the average frequency of markers associated with grain yield and its components exceeded 60%. For some favorable alleles, the frequency was 100%: *TaGS-D1b* and *ISBW2-GY* (T) in East Kazakhstan AES germplasm; *ISBW10-SM2* (G) in material from Karagandy AES, Kazakh Grain Center and Chelyabinsk Agricultural Research Institute (ARI).

**Figure 5:**
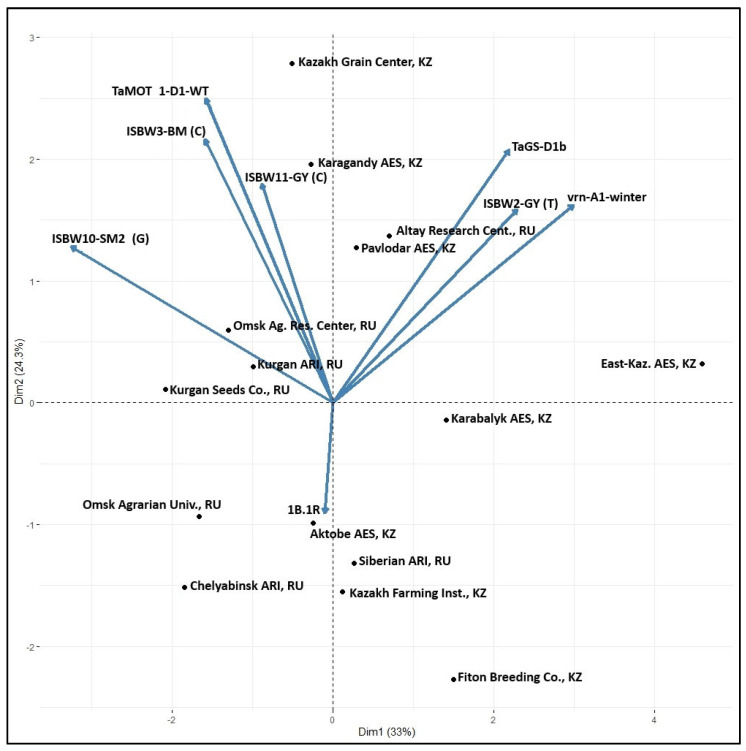
Biplot distribution of spring wheat breeding programs at 15 institutions according to the frequency of alleles at eight markers of agronomic traits (Table 4).

The important question was if the observed differences in the distribution of effective markers are related to agro-ecological conditions of the breeding programs or reflect the parentage, crossing and selection strategy employed by the breeders. At the time of the development of this germplasm, the breeders were not aware of the molecular markers and used conventional selection. The biplot location of the breeding programs according to the markers’ frequency (Figure 5) does not indicate the effect of agro-ecology as such. The breeding fields of the Omsk Agric. Research Center and University are only 5 km apart, but the markers’ frequency patterns are different. The same situation was found with Karabalyk AES and Fiton Breeding Company, and Kurgan ARI and Kurgan Seed Company. They are located close to each other, but the markers’ composition is different. There was a very close similarity between the breeding programs of Omsk Agrarian University and Kurgan Seeds, as well as Kazakh Grain Center and Karagandy ARI. East Kazakhstan AES stands apart from all other programs.

### 3.5. Identification of Superior Germplasm

A multilocational trial with 12 sites–years allowed the identification of superior high yielding germplasm using BLUEs. Due to the effect of maturity on grain yield, the highest yielding genotypes were identified separately for early (total of 24 genotypes), intermediate (96 genotypes) and late (22 genotypes) groups (Table 5 and Table S5). Four of the five highest yielding entries in the early group originated from the program in Russia including Siberian ARI in Novosibirsk (entries 27 and 29), Kurgan Seed (entry 127) and Samara ARI (entry 38). Lut. 166-SP94 was bred at Kazakh Farming Institute in Almaty, the most southern KASIB site. None of these lines possessed 1B.1R translocation and all carried the spring allele at the *Vrn-A1* locus. The presence of yield-related alleles varied depending on genotypes from four to six.

The top yielding cultivar in the intermediate group, Saratov-75, originated from South-East ARI in the Volga region. It did not possess 1B.1R translocation or the winter allele of the *Vrn-A1* locus. There are only five genotypes originating from Kazakhstan in the top twenty high yielding lines in this maturity group. The best Kazakh genotype, Lut. 1764, was ranked tenth. Five of the eight top-yielding genotypes possessed 1B.1R translocation. Four of the ten top-yielding genotypes possessed all five favorable alleles of markers *TaGS-D1b*, *ISBW10-SM2* (G), *ISBW2-GY* (T), *ISBW3-BM* (C), *ISBW11-GY* (C). This is, perhaps, another indirect indication of their efficiency.

The highest yielding cultivar in the late group and the whole set was Zauljbinka from East-Kazakhstan ARI, which possessed both 1B.1R translocation and the *vrn-A1* allele. The second and the third highest yielding genotypes in this group originated from Altay ARI. Cultivar Tobolskaya, which possessed all five favorable yield-related alleles, and it demonstrated good performance in KASIB regular trials and wide distribution in the production.

Considering the entire set of the best 30 genotypes, none of them possessed all eight favorable alleles. However, three breeding lines (146-Lut. 186/04-61, 49-Lut. 1764, 50-Lutescens 1082) combined seven of the eight favorable alleles.

## 4. Discussion

This is the first study in the KASIB region to assess the practical value of using a set of well-known molecular markers with respect to agronomic traits in a large set of diverse spring wheat germplasm using multi-locational trials with four sites and three seasons. The material used in the study represented 15 breeding programs, which cover over 80% of all spring wheat areas in Kazakhstan and Russia. Substantial phenotyping efforts allowed detailed characterization of the germplasm in contrasting conditions with significant genotype x environment interactions. Evaluation of the effects of molecular markers on agronomic traits led to the following general observations: (a) 17 out of 81 KASP markers evaluated in the germplasm were informative, the latter being excluded due to their unbalanced distribution or lack of phenotyping data; (b) eight markers (or 50%) of the set of informative markers proved to have a significant effect on the target traits; and (c) the effects of favorable alleles on agronomic traits was estimated to be in the range of 2–5%, but in combination, they can contribute to better performance; (d) the markers with significant effects in this study were developed outside of the KASIB region, demonstrating their universal nature.

The gene *Vrn-A1* has a large effect on vernalization requirements, diverse allelic variation [15] and contributes to genotype x environment interaction [16]. A previous study of 148 spring bread wheat cultivars in Western Siberia showed the presence of the *vrn-A1* allele at a frequency of 8% [17], whereas in this experiment it was also 8% in Russian material, but 35% in Kazakhstan germplasm. However, there were no previous reports on the effect of this allele on grain yield in the region.

*TaMOT-1-D1* is a marker related to the *Eps-D1* gene which accounts for the variation in flowering time when vernalization and photoperiod requirements are satisfied [18]. Askhadullin et al. [19] mention the importance of this gene for earliness in spring wheat cultivars from the Volga region. However, no data on the frequency or effects of this gene on earliness or yield in the KASIB region were available. The current study demonstrated that the late allele of this gene is widely spread in Kazakh and Russian germplasm and positively contributed to number of days to heading and grain yield.

The history of 1B.1R translocation and its use in wheat breeding is well described [20]. The translocation contributed to a successful breeding program at CIMMYT and was incorporated in global wheat germplasm [21]. The translocation contributed to yield potential [22] but also negatively affected the grain’s technological quality [23]. Korobkova et al. [24] documented the presence of 1B.1R translocation in over 50% of 66 modern winter wheat varieties from the Krasnodar breeding program. The frequency of 1B.1R translocation was over 25% in spring wheat stem rust resistance material studied in North Kazakhstan and Russia, as reported by Shamanin et al. [25]. The authors also mentioned a positive effect of 1B.1R translocation on grain yield. The current study proves the value of this translocation in the region with its pleiotropic effect on grain yield and stem rust resistance through the presence of the *Sr31* gene, affective against a wide range of pathotypes, including Ug99 [25].

The gene *TaGS-D1* on chromosome 7DS in wheat was described as ortholog to the rice gene *OsGS3* that plays a principal role in controlling grain weight and length in rice [26]. The study on European winter wheat did not show an effect of this marker on grain weight [27]; neither did a study of CIMMYT spring wheat [28]. Shan et al. [29] demonstrated its positive effect on yield components. So far, the gene’s effect had not been studied in Kazakhstan and Russia. Its frequency in the KASIB core set was around 70% and it increased both TKW and yield by 2–3%.

ICARDA identified several markers affecting yield and its components through association mapping in Sudan and Morocco. These markers include *ISBW2-GY* (*Kukri_c3243_1065*, 3B), *ISBW3-BM* (*TA004946-0577*, 1B), *ISBW10-SM2* (*BS00076246_51*, 5A), *ISBW11-GY* (*wsnp_Ex_c12812_20324622*, 4A) [30,31]. Despite the differences in agro-ecological conditions of North Africa and the KASIB region, these markers turned out to be effective in the current study, improving yield by 3–4%.

The distribution of a subset of eight effective markers in a large set of material across 15 breeding programs revealed large differences in their frequencies. These differences originate from the practical availability of diverse germplasm sources, from unconscious selection for optimal agronomic types, grain yield and resistance to biotic and abiotic stresses. However, the breeding programs’ choices of parents and selection strategy also contribute to this genetic diversity. Superior high-yielding genotypes were identified in all three maturity groups. Their multilocational characterization is supplemented by genetic composition for eight effective markers selected in this study. These superior genotypes can be used as parents for crossing programs.

What are the next steps in the utilization of molecular markers-assisted selection in practical breeding in KASIB institutions? The eight effective markers, including three with pleiotropic effects, identified in the current study have to be supplemented by SNP markers identified through GWAS on this diversity panel of spring wheat adapted to the region. Several studies have been completed, and results need to be converted to KASP markers for efficient use [12,13,14,15,16]. The KASIB core set GWAS for over 20 traits is underway and will contribute potentially useful markers. The simultaneous validation and use of existing and new markers need to include as many KASIB network sites as possible. Precision phenotyping and field trials will require the use of modern experimental design and statistical tools. Genotyping platforms in the region or efficient outsourcing would be an essential part of the markers’ use framework. Training of the breeders on the utilization of molecular markers, experimental design and phenotyping would be an important component of the successful application of genomic technologies in breeding.

## Figures and Tables

**Table 1:** The weather conditions of the experimental sites in 2020–2022 based on local meteo-stations.

Testing Site	Rainfall May–August, mm				Average Temperature in July, °C			
	2020	2021	2022	Long-Term	2020	2021	2022	Long-Term
Omsk, Omsk region	150	90	217	207	22.1	21.1	20.5	19.2
Karabalyk, Kostanay region	118	89	90	179	25.2	22.1	23.4	20.8
Shortandy, Akmola region	124	88	100	136	17.7	19.5	19.5	18.5
Shagalaly, North Kazakhstan	-	131	179	193	-	20.8	21.2	20.0

**Table 2:** Pearson coefficients of correlation between the BLUEs values for grain yield and agronomic traits across study sites, 2020–2022.

Site	Year	Coefficients of Correlation between the Grain Yield and:				
		Plant Height	Spike Length	Grains per Spike	1000 KW	Grain Weight per Spike
Karabalyk	2020	0.29 ***	0.01	0.40 ***	0.15	0.28 ***
	2021	-	0.03	0.39 ***	0.24 ***	0.44 ***
	2022	0.29 ***	−0.04	0.35 ***	0.19 **	0.23 ***
Omsk	2020	0.27 ***	0.19 **	0.40 ***	0.24 ***	0.41 ***
	2021	0.17 *	0.14	0.40 ***	0.11	0.35 ***
	2022	0.19 **	0.20 **	0.45 ***	0.25 ***	0.48 ***
Shagalaly	2021	0.49 ***	0.21 **	0.56 ***	0.30 ***	0.65 ***
	2022	0.44 ***	0.20 **	0.44 ***	0.32 ***	0.35 ***
Shortandy	2020	-	0.29 ***	0.38 ***	0.33 ***	0.38 ***
	2021	0.42 ***	0.26 ***	0.37 ***	0.25 ***	0.37 ***
	2022	0.51 ***	0.29 ***	0.36 ***	0.33 ***	0.48 ***
All	2020	0.83 ***	0.78 ***	0.81 ***	0.77 ***	0.82 ***
	2021	0.84 ***	0.75 ***	0.77 ***	0.63 ***	0.79 ***
	2022	0.77 ***	0.76 ***	0.79 ***	0.64 ***	0.79 ***

*, **, ***—significant at *p* < 0.05, 0.01 and 0.001, respectively.

**Table 3:** Effects of molecular markers on agronomic traits, BLUPs, 2020–2023.

Gene/Marker/Translocation	Trait	Number of Genotypes		Trait Average Value		Marker Effect ^1^, %	Number of Trials	
		−Marker	+Marker	−Marker	+Marker		Total	with Sign. Differences
*Rht8*	Plant height, cm	30	111	73.7 ± 0.9	74.3 ± 0.5	−0.8	9	0
*TaPpdDD002*	Days to heading	114	28	36.2 ± 0.2	36.7 ± 0.4	+1.4	6	1
*Vrn-B1*		98	44	36.0 ± 0.4	36.4 ± 0.2	−1.1	6	0
*PRR73-A1*		53	87	36.1 ± 0.2	36.6 ± 0.3	−1.4	6	0
*Vrn-A1*	Days to heading	28	113	35.9 ± 0.2	37.9 ± 0.4	−5.6 ^1^	6	6
	Yield, g/m^2^			225 ± 2	231 ± 3	−2.7 ^1^	11	6
*TaMOT1-D1*	Days to heading	70	61	35.8 ± 0.2	36.5 ± 0.3	−1.9 ^1^	6	5
	Yield, g/m^2^			221 ± 3	231 ± 2	−4.5 ^1^	11	8
*GS5-2334-SNP*	TKW, g	103	37	32.7 ± 0.2	32.9 ± 0.4	+0.6	11	0
	Yield, g/m^2^			223 ± 2	228 ± 3	+0.2	11	0
*TaGS-D1*	TKW	42	97	37.8 ± 0.3	38.6 ± 0.2	+2.1 ^1^	11	9
	Yield, g/m^2^			221 ± 4	228 ± 2	+3.2 ^1^	11	6
*ISBW10-SM2* ^1^	Total spikes/plant	28	114	2.05 ± 0.04 ^1^	2.21 ± 0.02 ^1^	+7.8 ^1^	3	3
	Prod. spikes/plant			1.63 ± 0.02 ^1^	1.70 ± 0.01 ^1^	+4.3 ^1^	5	3
	Yield, g/m^2^			222 ± 4	227 ± 2	+2.2	11	2
*TaCwi-4A*	Drought yield	113	26	226 ± 2	227 ± 3	−0.4	11	1
*Dreb1*	Drought yield	64	78	224 ± 3	229 ± 2	+2.2	11	1
1B.1R	Yield, g/m^2^	111	31	223 ± 2	237 ± 2	+6.2 ^1^	11	7
	Stem rust, %			27.6 ± 1.3 ^1^	6.6 ± 1.1 ^1^	−569.7 ^1^	1	1
*ISBW3-BM*	Yield, g/m^2^	97	44	220 ± 3	229 ± 3	+4.1 ^1^	11	9
*ISBW11-GY*	Yield, g/m^2^	54	86	221 ± 3	231 ± 2	+4.5 ^1^	11	8
*ISBW1-GY*	Yield, g/m^2^	31	111	222 ± 4	228 ± 2	−2.7	11	0
*ISBW2-GY*	Yield, g/m^2^	113	27	225 ± 2	233 ± 3	+3.5 ^1^	11	8
*IWAB8036*	Stem rust, %	67	75	22.6 ± 1.6 ^1^	23.4 ± 1.9 ^1^	−3.4	1	0

^1^—Significant differences between the groups of germplasm with and without favorable alleles.

**Table 4:** Distribution of eight molecular markers of agronomic traits in the germplasm from KASIB breeding programs.

Breeding Program	Number of Genotypes Evaluated	Favorable Allele Frequency (%) for Target Traits:								
		Days to Heading		Yield, Stem Rust	TKW, Yield	Spike Number, Yield	Biomass, Yield	Yield		All
		*TaMOT 1-D1*	*vrn-A1*	1B.1R	*TaGS-D1b*	*ISBW10-SM2* (G)	*ISBW3-BM* (C)	*ISBW11-GY* (C)	*ISBW2-GY* (T)	Average
Kazakhstan										
Aktobe AES	22	20.0	0	4.5	86.4	77.3	81.8	54.5	68.2	49.1
East Kaz. AES	21	33.3	95.0	47.6	100.0	23.8	42.9	80.0	100.0	65.3
Fiton Breeding Co.	16	23.1	18.8	31.3	62.5	62.5	50.0	25.0	93.3	45.8
Karabalyk AES	25	45.8	40.0	4.2	84.0	62.5	56.0	56.0	84.0	54.1
Karagandy AES	36	65.7	41.7	0.0	88.6	100.0	97.2	52.8	86.1	66.5
Kazakh Farm. Inst.	28	33.3	10.7	3.6	53.6	67.9	64.3	46.4	82.1	45.2
Kazakh Grain Cent.	14	71.4	28.6	0.0	92.9	100.0	92.9	85.7	92.9	70.6
Pavlodar AES	25	70.8	45.8	0.0	72.0	84.0	80.0	50.0	92.0	61.8
Total/average	187	45.4	35.1	11.4	80.0	72.3	70.6	56.3	87.3	57.3
Russia										
Altay Res. Cent.	27	69.2	34.6	3.7	86.4	85.2	51.9	74.1	96.3	62.7
Chelyabinsk ARI	14	53.8	0.0	21.4	41.7	100.0	35.7	78.6	69.2	50.1
Kurgan ARI	21	35.0	4.8	14.3	57.1	95.2	85.7	85.7	92.9	58.8
Kurgan Seeds Co.	15	71.4	7.1	57.1	71.4	93.3	73.3	86.7	60.0	65.0
Siberian ARI	15	47.4	0.0	21.1	78.9	68.4	52.6	42.1	77.8	48.5
Omsk Agr. Univ.	22	57.1	4.5	27.3	45.5	86.4	71.4	63.6	71.4	53.4
Omsk Res. Cent.	21	70.0	4.8	57.1	61.9	90.5	90.5	66.7	90.5	66.5
Total/average	135	57.7	8.0	28.9	63.3	88.4	65.9	71.1	79.7	57.9

**Table 5:** Grain yield, number of days to heading, TKW and presence of the effective markers in the top yielding germplasm.

#	Genotype	Grain Yield ^1^, g/m^2^	Days to Head. ^2^	TKW ^1^, g	*1B.1R*	*TaMOT 1-D1-Ria*	*vrn-A1*	*TaGS-D1b*	*ISBW* *10-SM2-G*	*ISBW* *2-GY-C*	*ISBW* *3-BM-C*	*ISBW* *11-GY-T*
Early heading group (35–39.9 days)												
27	*Novosib.18*	273	39.6	34.3	−	−	−	+	+	−	−	−
78	*Lut. 166-CΠ94*	272	39.5	42.7	−	+	−	+	+	+	+	+
127	*Lut. KS 963*	270	39.8	33.6	−	−	−	+	+	−	+	+
38	*Lut.1193*	267	39.3	34.1	−	−	−	+	+	+	−	+
52	*Lut.2102*	263	39.7	38.1	−	+	−	+	+	−	+	−
Intermediate heading group (40–44 days)												
41	*Saratov. 75*	317	40.8	37.5	−	−	−	+	+	−	+	−
110	*Lut.37-17*	313	43.7	40.3	+	−	−	+	+	+	+	+
36	*Ekada 113*	311	42.5	39.5	−	+	−	+	+	+	+	−
126	*Line-241-00-4*	306	43.3	38.1	+	+	−	−	+	+	−	+
130	*Erythrosp. 78*	305	40.5	34.8	−	+	−	−	+	−	+	+
146	*Lut.186/04-61*	302	43.5	39.0	+	+	−	+	+	+	+	+
49	*Lut.1764*	300	43.0	38.4	−	+	+	+	+	+	+	+
132	*Lut.126-05*	299	41.2	36.7	−	+	−	−	+	−	+	+
70	*Samgau*	297	42.2	40.6	−	−	−	+	+	−	−	−
28	*Lut. 307/97-23*	297	43.6	36.8	+	+	−	+	−	+	+	+
148	*SPChS 69*	296	43.0	39.0	−	+	−	+	+	+	−	−
50	*Lut.1082*	296	43.4	40.0	−	+	+	+	+	+	+	+
116	*OK-1*	293	41.8	39.9	+	−	−	+	+	+	+	+
112	*Lut.106-0/2003*	293	42.2	36.3	−	+	+	−	−	+	+	−
68	*Lutescens 9-33*	290	42.0	37.2	−	+	+	−	−	+	+	−
115	*A-125*	290	41.0	35.2	−	+	−	+	+	+	−	−
18	*GVK 1857/9*	288	43.7	39.2	−	+	+	+	−	+	−	+
107	*Chebarkul.*	288	42.1	38.7	−	−	−	+	+	+	−	+
1	*Altay. zhnitsa*	288	41.9	37.7	−	+	−	+	+	+	−	+
Late heading group (44–48 days)												
21	*Zauljbinka*	327	44.5	35.7	+	−	+	+	−	+	−	+
6	*Tobol. 1*	302	44.8	38.4	−	+	−	+	+	+	+	+
7	*Lut.1012*	301	44.5	37.9	−	+	−	+	+	+	−	+
69	*Pavlodar. 11*	298	44.3	34.6	−	−	−	−	+	+	+	−
40	*LD-25*	293	45.5	37.8	−	+	+	+	+	+	+	+

^1^—BLUEs across all trials; ^2^—average values for original data.

## Data Availability

Data are available from the corresponding author upon request.

## Supplementary Materials

The following supporting information can be downloaded at: https://www.mdpi.com/article/10.3390/genes15010086/s1, Table S1. The list of materials used in the study; Table S2. The list of KASP markers used in the study; Table S3. Grain yield and agronomic traits at three experimental sites in 2020–2022; Table S4. ANOVA results for grain yield and agronomic traits at three experimental sites in 2020–2022; Table S5. Grain yield, number of days to heading, TKW and presence of effective molecular markers in KASIB set.
