# Supplementary material for: Molecular Markers Help with Breeding for Agronomic Traits of Spring Wheat in Kazakhstan and Siberia
Source: Genes (Basel). 2024 Jan 10;15(1):86. doi: 10.3390/genes15010086 (PMC10815559; doi:10.3390/genes15010086)
Supplement: Supplementary file 1 [file genes-15-00086-s001.zip › genes-2636960-supplementary.pdf]

Table S1. The list of material used in the study.

Entry #	Germplasm name	Origin
1	Altayskaya zhnitsa	Altay ARI, RU
2	Altayskaya 530	Altay ARI, RU
3	Sibirskiy Aljans	Altay ARI, RU
4	Lutescens 844	Altay ARI, RU
5	Stepnaya volna	Altay ARI, RU
6	Toboljskaya-1	Altay ARI, RU
7	Lutescens 1012	Altay ARI, RU
8	Lut.509	Altay ARI, RU
9	Lutescens 509	Altay ARI, RU
10	Lutescens 1003	Altay ARI, RU
11	Erythrospermum 1119	Altay ARI, RU
12	Lutescens 574	Altay ARI, RU
13	Lutescens 697	Altay ARI, RU
14	Lutescens 665/1	Altay ARI, RU
15	Lutescens 424	Altay ARI, RU
16	Lutescens 716	Altay ARI, RU
17	Velyutinum 15	East-Kaz.stan ARI, KZ
18	GVK 1857/9	East-Kaz.stan ARI, KZ
19	GVK 1860-12	East-Kaz.stan ARI, KZ
20	Lyazzat	East-Kaz.stan ARI, KZ
21	Zauljbinka	East-Kaz.stan ARI, KZ
22	Novosibirskaya 31	Siberian ARI, RU
23	Novosibirskaya 16	Siberian ARI, RU
24	Sibirskaya 21	Siberian ARI, RU
25	Novosibirskaya 15	Siberian ARI, RU
26	Novosibirskaya 29	Siberian ARI, RU
27	Novosibirskaya 18	Siberian ARI, RU
28	Lutescens 307/97-23	Siberian ARI, RU
29	L. 196/94-6	Siberian ARI, RU
30	Omskaya 37	Siberian ARI, RU
31	Aktobe 1580	Aktobe AES, KZ
32	Asap	Aktobe AES, KZ
33	Stepnaya 259	Aktobe AES, KZ
34	Stepnaya 1509/06	Aktobe AES, KZ
35	Stepnaya 62	Aktobe AES, KZ
36	Ekada 113	Ekada, RU
37	Ekada 85	Ekada, RU
38	Lutescens 1193	Samara ARI, RU
39	L-654	South-East ARI, RU
40	LD-25	South-East ARI, RU
41	Saratovskaya 75	South-East ARI, RU
42	Saratovskaya 29-2	South-East ARI, RU
43	Karagandinskaya-93	Karagandy AES, KZ
44	Lutescens - 1212	Karagandy AES, KZ

45	Lutescens - 1226	Karagandy AES, KZ
46	Lutescens - 1235	Karagandy AES, KZ
47	Sary Arka 28 (Лют.1599)	Karagandy AES, KZ
48	Lutescens 1519	Karagandy AES, KZ
49	Lutescens 1764	Karagandy AES, KZ
50	Lutescens1082	Karagandy AES, KZ
52	Lutescens 2102	Karagandy AES, KZ
53	Lutescens 1350	Karagandy AES, KZ
54	Lutescens 1501	Karagandy AES, KZ
55	Lutescens - 1135	Karagandy AES, KZ
56	Tselinnaya niva	Kaz. Grain Center, KZ
57	Astana 2	Kaz. Grain Center, KZ
58	Lutescens 230/00	Kaz. Grain Center, KZ
59	Shortandinskaya 95 uluchshennaya	Kaz. Grain Center, KZ
60	Astana	Kaz. Grain Center, KZ
61	Tselina 50	Kaz. Grain Center, KZ
64	27-90-98-3	Pavlodar ARI, KZ
65	53-88-94-12	Pavlodar ARI, KZ
66	53-94-98-2	Pavlodar ARI, KZ
67	Lutescens 53/95-98-1	Pavlodar ARI, KZ
68	Lutescens 9-33	Pavlodar ARI, KZ
69	Pavlodarskaya 11	Pavlodar ARI, KZ
70	Samgau	Kaz. Farming Inst.
71	Aray	Kaz. Farming Inst., KZ
72	Iljinskaya	Kaz. Farming Inst., KZ
73	Lutescens - 70	Kaz. Farming Inst., KZ
74	Yrym	Kaz. Farming Inst., KZ
75	Lutescens 24	Kaz. Farming Inst., KZ
76	Lutescens 715-04	Kaz. Farming Inst., KZ
77	Erythrospermum 727	Kaz. Farming Inst., KZ
78	Lutescens 166-СП94	Kaz. Farming Inst., KZ
81	Kayir G-5454-91	Kaz. Farming Inst., KZ
82	Kaz.stanskaya-3	Kaz. Farming Inst., KZ
83	Nadezhnda	Kaz. Farming Inst., KZ
84	Fiton 109	Fiton KZ
85	Fiton C 36 ЧС	Fiton KZ
86	Fiton 43	Fiton KZ
87	Fiton C 50 ЧС	Fiton KZ
88	Ekada 148	Fiton KZ
89	Fiton C-54	Fiton KZ
90	Fiton 25	Fiton KZ
91	Fiton 41	Fiton KZ
92	Fiton 156	Fiton KZ
93	Fiton C 41 ЧС	Fiton KZ
96	Lutescens C 19 ЧС	Karabalyk AES, KZ
97	Lutescens 36	Karabalyk AES, KZ
98	Avgustina	Karabalyk AES, KZ
99	Liniya-22 ЧС	Karabalyk AES, KZ

100	Liniya-4-10-16	Karabalyk AES, KZ
101	Lutescens 48-204-03	Karabalyk AES, KZ
102	Lutescens 4	Karabalyk AES, KZ
103	Ayna	Karabalyk AES, KZ
104	Chelyaba yubileinaya	Chelyabinsk ARI, RU
105	Lutescens 23490	Chelyabinsk ARI, RU
106	Silach	Chelyabinsk ARI, RU
107	Chebarkuljskaya	Chelyabinsk ARI, RU
108	Pamyati Ryuba	Chelyabinsk ARI, RU
109	Lutescens 22-17	Kurgan ARI, RU
110	Lutescens 37-17	Kurgan ARI, RU
111	P-23-14	Kurgan ARI, RU
112	Lutescens 106-0/2003	Kurgan ARI, RU
113	Lutescens 120/2003	Kurgan ARI, RU
114	SAD-101	Kurgan ARI, RU
115	A-125	Kurgan ARI, RU
116	OK-1	Kurgan ARI, RU
117	Alfa 79	Kurgan ARI, RU
118	Lutescens 8-108-1	Kurgan ARI, RU
119	Lutescens 363/96-4	Kurganseeds, RU
120	Lutescens 360/96-6	Kurganseeds, RU
121	Lutescens 205/03-1	Kurganseeds, RU
123	Lutescens KS 14/09-2	Kurganseeds, RU
124	Lutescens KS 140/08-3	Kurganseeds, RU
125	Lutescens 290/99-7	Kurganseeds, RU
126	Liniya-241-00-4	Kurganseeds, RU
127	Lutescens KS 963	Kurganseeds, RU
128	Lutescens 128-05	Omsk State Agr. Univ., RU
129	Erythrospermum 85-08	Omsk State Agr. Univ., RU
130	Erythrospermum 78	Omsk State Agr. Univ., RU
131	Lutescens 89-06	Omsk State Agr. Univ., RU
132	Lutescens 126-05	Omsk State Agr. Univ., RU
133	Lutescens 96-12	Omsk State Agr. Univ., RU
134	OmGAU-100	Omsk State Agr. Univ., RU
135	Stolypinskaya 2	Omsk State Agr. Univ., RU
136	Chernyava 13	Omsk State Agr. Univ., RU
137	Sibakovskaya Yubilienaya	Omsk State Agr. Univ., RU
138	Lutescens 27-12	Omsk State Agr. Univ., RU
139	Tertsiya-1	Omsk State Agr. Univ., RU
140	Lutescens 7/04-26	Omsk Agr. Res. Center, RU
141	Lutescens 141/03-2	Omsk Agr. Res. Center, RU
142	Lut. 3/04-21-11	Omsk Agr. Res. Center, RU
143	Lutescens 529/00-10C	Omsk Agr. Res. Center, RU
144	Pamyati Azieva	Omsk Agr. Res. Center, RU
145	Sigma	Omsk Agr. Res. Center, RU
146	Lutescens 186/04-61	Omsk Agr. Res. Center, RU
147	Lutescens 6/04-4	Omsk Agr. Res. Center, RU
148	SPChS 69	Omsk Agr. Res. Center, RU

149	Omskaya 35-2	Omsk Agr. Res. Center, RU
150	Omskaya 41	Omsk Agr. Res. Center, RU

Table S2. The list of KASP markers used in the study.

#	Gene	Marker name	Allele effect
**Development rate**			
1	*Ppd-A1*	*GS105-1117ID*	Ppd-A1b: Photoperiod insensitive
2*	*PRR73-A1*	*PRR73A1-9IND*	Hap-II: Late flower
3	*PRR73-B1*	*PRR73B1-4558*	Hap-II: Early flower
4	*TaELF3-D1*	*FT3-Exon4_A/G*	Savanah-type: Early flower
5*	*TaMOT1-D1*	*TaMOT1-D1_KASP1*	Wild-type: Early flower
6	*Ppd-B1*	*TaPpdBJ001*	Ppd-B1b: Photoperiod sensitive
7	*Ppd-D1*	*TaPpdDD001*	Ppd-D1b: Photoperiod sensitive (Late flowering)
8*	*Ppd- D1*	*TaPpdDD002*	Del: Early flower
9*	*Vrn-A1*	*Vrn1_new*	Vrn-A1a spring: Spring-type early flower
10*	*Vrn-A1*	*Vrn-A1_9K0001*	2147-type: Long vern
11*	*Vrn-A1*	*Exon7_C/T_Vrn-A1*	Hereward-type: Late flower
12*	*Vrn-B1*	*wMAS000037*	Vrn-B1a-c: Early flower
13*	*Vrn-D1*	*Vrn-D1-D1a_A*	vrn-D1: Late flower
**Disease resistance**			
14	*Excalibur_c7282_512*	*Excalibur_c7282_512*	A: Fhb sus
15	*Fhb1*	*Fhb1_KSU*	Fhb1- : Fhb1 gene absent
16*	*QSr.icarda-7A.1*	*IWAB8036*	A: Ug99 res
17	*QSr.icarda-7A.2*	*IWB30995*	C: Ug99 res
18	*QSr.icarda-7A.3*	*IWB46162*	T: Ug99 sus
19*	*QSr.icarda-7A.4*	*IWB58668*	G: Ug99 res
20	*Kukri_c36639_186*	*Kukri_c36639_186*	Fusarium head blight
21*	*Lr34*	*Lr34_TCCIND*	Lr34-: Lr34 gene absent
22	*Lr37*	*VPM_SNP*	Lr37-: Lr37 gene absent
23*	*Lr46*	*Lr46_JF2-2A*	Lr46-: Lr46 gene absent
24	*Lr47*	*Lr47*	Lr47-: Lr47 gene absent
25	*Lr67*	*TM4_67*	Lr67-: Lr67 gene absent
26*	*Lr68*	*Lr68-2*	Lr68-: Lr68 gene absent
27	*Lr9*	*Wsnp1Lr9*	Lr9-: Lr9 gene absent
28	*Pch1*	*wMAS000023*	Pch1-: Eye spot resistance gene absent
29	*Sbm1*	*wMAS000016*	Sbm1-: Soil born mosaic virus gene absent
30	*SrCad*	*snpTA0035*	SrCad, Sr42, SrTmp-: Absent
31	*Sr2*	*wMAS000005*	Sr2-: Sr2 gene absent

32	*Sr36*	*wMAS000015*	Sr36-: Sr36 gene absent
33	*Tsn1*	*Tsn1*	Tsn1-: Tan spot resistance gene absent
34	*Yr5*	*Wsnp1Yr5*	Yr5
35	*Yr57*	*BS00062676*	Yr57-: Yr57 gene absent
36	*Zds-A1*	*Zds-A1*	TaZds-A1a: Low YPC
**Grain quality**			
37	*GCP*	*GCP_DUP*	Gpc-B1-: Low grain protein contents
38	*Glu-A1*	*gluA1.1_1883_ALA*	Null: Low quality
39	*Glu-D1*	*Glu-D1d_SNP*	2+12: Weak gluten
40	*Pinb-D1*	*Pinb-D1_INS*	Pinb-D1b: Hard
41	*PPO-D1*	*PPO-D1_SNP*	Ppo-D1a: Low PPO
Height			
42*	*Rht8*	*Rht8*	Rht8a: Tall
43	*Rht-B1*	*wMAS000001*	Rht-B1b: Short
44	*Rht-D1*	*Rht-D1_SNP*	Rht-D1b: Short
Insect resistance			
45	*QNr.icarda-7B*	*Bobwhite_rep_c66630_331*	A: Nematode res
46	*Ei1*	*BS00022785*	T: Sunn pest sus
47	*Cre8*	*Cre8_SNP*	Cre8-: Soil born disease gene absent
48	*QNr.icarda-2B*	*Excalibur_c18966_804*	A: Nematode sus
49	*QNr.icarda-5B.1*	*Excalibur_c78724_434*	A: Nematode res
50	*Ei1*	*IWB66138*	G: Sunn pest res
51	*Rlnn1*	*Rlnn1*	Rlnn-: Root leision nematode gene absent
52	*QNr.icarda-5B.2*	*Tdurum_contig10380_87*	A: Nematode res
53	*QNr.icarda-4A.1*	*Tdurum_contig82236_117*	A: Nematode res
54	*QNr.icarda-3A*	*wsnp_BE426418A_Ta_2_1*	C: Nematode sus
55	*QNr.icarda-1A*	*wsnp_BE443588A_Ta_2_1*	G: Nematode sus
56	*QNr.icarda-4A.2*	*wsnp_Ex_c55245_57821389*	C: Nematode res
**Morphology**			
57	*QAwns.icarda-5AL*	*BobWhite_C8266_227_TG_5AL*	G: Awned
58	*Tamby10-A1*	*Tamby10-A1*	R-A1b: Red grain color allele
59	*Tamyb-B1*	*TamybR B1a-b_C*	R-B1b: Red grain color allele
**Yield**			
60*	*1B.1R*	*wMAS000011*	1B.1R: rye translocation
61*	*Dreb-B1*	*Dreb1_JC_3BL*	TaDREB-B1b: Drought susceptible allele
62*	*QYld.icarda-4A*	*ISBW11-GY,* *wsnp_Ex_c12812_20324622*	T: Lower yield

63*	*QYld.icarda-5A*	*ISBW1-GY, wsnp_Ex_c2526_4715978*	G: Higher yield
64*	*QYld.icarda-3B*	*ISNW2-GY, Kukri_c3243_1065*	C: Lower yield
65*	*TaCwi-4A*	*TaCwi-4A*	Hap-4A-T: Low yield under drought
66	*TaCwi-5D*	*TaCwi-5D*	Hap-5D-G: Low yield iunder drought
**Yield components**			
67*	*QSm2.icarda-5A*	*ISBW10-SM2, BS00076246_51*	G: Higher spike number
68*	*QBm.icarda-1B.1*	*ISBW3-BM, TA004946-0577*	C: Higher biomass
69	*QBm.icarda-1B.2*	*ISBW4-BM, Excalibur_c11980_619*	T: Higher biomass
70*	*QGps.icarda-4A*	*ISBW7-GPS, wsnp_Ex_rep_c66324_64493429*	T: Higher grains per spike
71*	*QSm2.icarda-5B*	*ISBW9-SM2, Excalibur_c71712_180*	T: Higher spike number
**TKW**			
72*	*TaGS5-A1*	*GS5-2334-SNP*	TaGS5-A1b: High grain size
73	*TaGW2-6B*	*GW2-6B*	Hap-III: Low TGW
74	*QTkw.icarda-3A*	*IBSW5-TKW, BS00057445_51*	G: Lower TKW
75	*TaSus2-2B*	*wMAS000021*	Hap-H: High TGW
76*	*TaGS-D1*	*TaGS-D1*	TaGD-D1b
77	*TaSus1-7B*	*Sus1-7B-2932IND*	Hap-C: Low TGW
78	*TaSus2-2A*	*TaSus2-2A*	Hap-A: High TGW
79	*TaSus-7A*	*TaSus-7A*	TaSus-7A-2: High TGW
80*	*TEF-7A*	*TEF-7A*	Hap-7A-1, 2: Lower TKW
81	*TPP-6A_AL1*	*TPP-6A_AL1*	TPP-6AL1b: Low TGW

* - markers used in the study.

Table S3. Grain yield and agronomic traits at three experimental sites in 2020-2022.

Site	Year	Plant height, cm	Spike length, cm	Grains per spike	Grain weight per spike, g	1000 kernel weight, g	Grain yield, g/m^2
Omsk State Agrarian University	2020	91.5	9.3	36.2	1.52	41.9	366
	2021	89.5	10.0	35.5	1.40	39.5	569
	2022	87.1	9.2	35.7	1.51	42.1	388
	Mean (21-22)	88.3	9.6	35.6	1.46	40.8	478
Karabalyk Agric. Exp. Station	2020	69.7	8.1	28.6	1.01	35.4	165
	2021	-	6.0	23.7	0.75	29.8	118
	2022	75.2	7.4	27.3	1.05	33.2	239
	Mean (21-22)	72.5	6.7	25.5	0.90	31.5	178
Kaz. Grain Res. Center	2020	-	7.3	26.6	0.91	35.0	161
	2021	65.1	7.3	28.2	0.93	35.6	183
	2022	56.4	6.6	25.3	0.78	35.9	248
	Mean (21-22)	60.8	7.0	26.8	0.86	35.8	215
North Kaz. AES	2021	58.5	8.0	26.4	1.02	36.8	163
	2022	74.5	7.1	21.8	1.16	41.3	336
	Mean (21-22)	66.5	7.6	24.1	1.09	39.1	249

Table S4. ANOVA results for grain yield and agronomic traits at three experimental sites in 2020-2022.

Main effects and interactions	Probability values for main effects and interaction for the following traits:					
	Plant height	Spike length	Grains per spike	Grain weight per spike	1000 kernel weight	Grain yield
Genotype	<0.001	<0.001	<0.001	<0.001	<0.001	<0.001
Site	<0.001	<0.001	<0.001	<0.001	<0.001	<0.001
Year	<0.001	<0.001	<0.001	<0.001	<0.001	<0.001
Genotypes x Sites	<0.001	<0.001	<0.001	<0.001	<0.01	<0.001
Genotypes x Years	0.638	0.165	0.433	0.381	<0.001	0.572
Sites x Years	<0.001	<0.001	<0.001	<0.001	<0.001	<0.001

Table S5. Grain yield (BLUEs), number of days to heading (original data), TKW (BLUEs)and presence of effective molecular markers in KASIB core set.

№	VARIETY-ENG	ORIGIN-INSTITUTE	Grain yield, g/m²	Days to heading	TKW, g	1B.1R	TaMOT1-D1-Ria	vrn-A1	TaGS-D1	ISBW10-SM2-G	ISBW2-GY-C	ISBW3-BM-C	ISBW11-GY-T
Early heading group (35-39.9 days)													
27	Novosibirskaya 18	Novosibirsk ARI	273	39.6	34.3	-	-	-	+	+	-	-	-
78	Lutescens 166-СП94	Kaz. Farming Inst.	272	39.5	42.7	-	+	-	+	+	+	+	+
127	Lutescens KS 963	Kurganseeds	270	39.8	33.6	-	-	-	+	+	-	+	+
38	Lutescens 1193	Samara ARI	267	39.3	34.1	-	-	-	+	+	+	-	+
52	Lutescens 2102	Karagandy ARI	263	39.7	38.1	-	+	-	+	+	-	+	-
54	Lutescens 1501	Karagandy ARI	260	39.8	34.8	-	-	-	-	+	+	+	-
31	Aktobe 1580	Aktobe AES	258	39.8	41.3	-	-	-	+	+	+	+	+
60	Astana	Kaz. Grain Inst.	254	39.9	32.8	-	+	-	+	+	-	+	+
144	Pamyati Azieva	Siberian ARI	253	39.1	36.0	-	-	-	+	+	+	+	+
91	Fiton 41	Fiton	252	38.9	39.2	-	-	-	-	+	n.a.	-	-
135	Stolypinskaya 2	Omsk SAU	248	39.8	39.4	-	n.a.	-	+	+	+	+	+
42	Saratovskaya 29-2	South-East ARI	246	38.8	36.2	-	+	-	+	+	+	+	+
20	Lyazzat	East-Kaz. ARI	245	39.9	36.3	-	-	-	+	+	+	+	n.a.
71	Aray	Kaz. Farming Inst.	236	38.7	37.3	-	+	-	-	-	+	+	+
92	Fiton 156	Fiton	234	39.3	35.1	-	-	-	+	-	+	+	-
25	Novosibirskaya 15	Novosibirsk ARI	229	35.0	32.7	-	+	-	+	+	+	+	-
32	Asap	Aktobe AES	227	37.8	37.0	-	-	-	+	+	+	+	+
29	L. 196/94-6	Novosibirsk ARI	225	38.5	40.5	-	-	-	+	+	-	+	+
73	Lutescens - 70	Kaz. Farming Inst.	220	38.0	39.5	-	-	-	+	+	+	-	-

111	P-23-14	Kurgan ARI	218	39.3	33.1	-	+	-	-	+	+	+	-
117	Alfa 79	Kurgan ARI	211	36.3	35.1	-	-	-	+	+	+	+	n.a.
26	Novosibirskaya 29	Novosibirsk ARI	208	38.4	34.1	-	+	-	+	+	+	+	-
74	Yrym	Kaz. Farming Inst.	197	38.5	36.1	-	-	-	-	-	+	-	-
23	Novosibirskaya 16	Novosibirsk ARI	188	35.0	34.5	-	+	-	+	+	+	-	-
Intermediate heading group (40-44 days)													
41	Saratovskaya 75	Saratov ARI	317	40.8	37.5	-	-	-	+	+	-	+	-
110	Lutescens 37-17	Kurgan ARI	313	43.7	40.3	+	-	-	+	+	+	+	+
36	Ekada 113	Ekada	311	42.5	39.5	-	+	-	+	+	+	+	-
126	Liniya-241-00-4	Kurganseeds	306	43.3	38.1	+	+	-	-	+	+	-	+
130	Erythrospermum 78	Omsk SAU	305	40.5	34.8	-	+	-	-	+	-	+	+
146	Lutescens 186/04-61	Siberian ARI	302	43.5	39.0	+	+	-	+	+	+	+	+
49	Lutescens 1764	Karagandy ARI	300	43.0	38.4	-	+	+	+	+	+	+	+
132	Lutescens 126-05	Omsk SAU	299	41.2	36.7	-	+	-	-	+	-	+	+
70	Samgau	Kaz. Farming Inst.	297	42.2	40.6	-	-	-	+	+	-	-	-
28	Lutescens 307/97-23	Novosibirsk ARI	297	43.6	36.8	+	+	-	+	-	+	+	+
148	SPChS 69	Siberian ARI	296	43.0	39.0	-	+	-	+	+	+	-	-
50	Lutescens1082	Karagandy ARI	296	43.4	40.0	-	+	+	+	+	+	+	+
116	OK-1	Kurgan ARI	293	41.8	39.9	+	-	-	+	+	+	+	+
112	Lutescens 106-0/2003	Kurgan ARI	293	42.2	36.3	-	+	+	-	-	+	+	-
68	Lutescens 9-33	Pavlodar ARI	290	42.0	37.2	-	+	+	-	-	+	+	-
115	A-125	Kurgan ARI	290	41.0	35.2	-	+	-	+	+	+	-	-
18	GVK 1857/9	East-Kaz. ARI	288	43.7	39.2	-	+	+	+	-	+	-	+

107	Chebarkuljskaya	Chelyabinsk ARI	288	42.1	38.7	-	-	-	+	+	+	-	+
1	Altayskaya zhnitsa	Altay ARI	288	41.9	37.7	-	+	-	+	+	+	-	+
131	Lutescens 89-06	Omsk SAU	287	41.6	36.3	-	+	-	-	+	-	+	-
119	Lutescens 363/96-4	Kurganseeds	286	43.7	38.2	+	+	-	+	+	+	+	+
118	Lutescens 8-108-1	Kurgan ARI	286	42.6	34.8	-	n.a.	-	+	+	-	+	-
123	Lutescens KS 14/09-2	Kurganseeds	286	43.4	38.0	+	-	-	-	+	-	+	+
33	Stepnaya 259	Aktobe AES	283	41.6	37.5	-	-	-	-	+	+	+	-
102	Lutescens 4	Karabalyk AES	283	43.0	36.4	-	+	+	-	-	+	+	-
106	Silach	Chelyabinsk ARI	280	43.9	39.5	+	+	-	+	+	+	+	+
129	Erythrospermum 85-08	Omsk SAU	279	43.6	36.2	+	+	-	+	+	n.a.	+	+
57	Astana 2	Kaz. Grain Inst.	278	40.5	39.0	-	+	-	+	+	+	+	-
125	Lutescens 290/99-7	Kurganseeds	278	43.9	38.9	+	+	-	-	+	+	+	+
58	Lutescens 230/00	Kaz. Grain Inst.	278	42.9	39.4	-	+	-	+	+	+	+	+
47	Sary Arka 28 (Лют.1599)	Karagandy ARI	278	43.1	36.3	-	+	+	-	+	+	+	+
113	Lutescens 120/2003	Kurgan ARI	276	40.3	37.0	-	+	-	+	+	+	+	+
48	Lutescens 1519	Karagandy ARI	276	42.7	40.4	-	+	+	+	+	+	+	+
77	Erythrospermum 727	Kaz. Farming Inst.	275	43.9	38.7	-	+	+	-	-	+	+	+
140	Lutescens 7/04-26	Siberian ARI	275	43.5	36.7	+	+	-	-	+	-	+	+
138	Lutescens 27-12	Omsk SAU	274	42.8	39.1	-	+	-	+	+	+	-	-
14	Lutescens 665/1	Altay ARI	274	41.8	36.5	-	+	n.a.	+	+	-	-	+
109	Lutescens 22-17	Kurgan ARI	273	43.3	39.3	+	-	-	-	+	+	+	+
43	Karagandinskaya-93	Karagandy ARI	273	40.8	36.1	-	+	-	+	+	+	+	+
65	53-88-94-12	Pavlodar ARI	272	41.0	36.1	-	-	-	+	+	+	+	+

61	Tselina 50	Kaz. Grain Inst.	271	40.2	36.7	-	+	-	+	+	+	+	-
64	27-90-98-3	Pavlodar ARI	269	40.3	37.8	-	n.a.	-	-	+	-	+	-
85	Fiton C 36 ЧС	Fiton	269	42.0	40.4	-	-	-	+	+	+	-	-
66	53-94-98-2	Pavlodar ARI	269	40.9	37.9	-	+	-	+	+	+	+	+
114	SAD-101	Kurgan ARI	269	41.9	37.3	-	-	-	+	+	+	+	+
88	Ekada 148	Fiton	266	42.4	38.0	-	n.a.	-	+	+	-	+	-
35	Stepnaya 62	Aktobe AES	265	40.3	37.3	-	n.a.	-	-	+	-	+	-
124	Lutescens KS 140/08-3	Kurganseeds	265	42.6	36.4	+	+	-	+	+	-	+	+
19	GVK 1860-12	East-Kaz. ARI	264	43.5	36.2	-	+	+	+	-	+	-	+
59	Shortandinskaya 95 uluchshennaya	Kaz. Grain Inst.	263	43.0	39.2	-	+	-	+	+	+	+	+
101	Lutescens 48-204-03	Karabalyk AES	263	41.4	38.8	-	-	-	+	+	+	-	+
24	Sibirskaya 21	Novosibirsk ARI	262	40.9	36.3	-	-	-	+	+	+	-	-
147	Lutescens 6/04-4	Siberian ARI	261	43.9	38.4	+	+	-	+	+	+	-	+
37	Ekada 85	Ekada	261	40.6	31.3	-	+	+	+	+	+	-	+
90	Fiton 25	Fiton	260	42.8	38.3	+	-	-	+	+	+	-	-
45	Lutescens - 1226	Karagandy ARI	259	41.0	38.7	-	-	+	+	+	+	+	+
30	Omskaya 37	Novosibirsk ARI	259	42.0	36.2	+	+	-	+	+	-	+	+
15	Lutescens 424	Altay ARI	258	40.6	37.9	-	-	+	+	-	+	+	+
5	Stepnaya volna	Altay ARI	258	40.9	39.3	-	-	-	-	+	+	-	-
98	Avgustina	Karabalyk AES	257	43.3	41.0	-	+	+	+	-	-	+	+
84	Fiton 109	Fiton	257	40.0	38.1	-	-	-	+	+	+	+	-
128	Lutescens 128-05	Omsk SAU	257	41.6	33.8	-	-	-	-	+	-	+	+
143	Lutescens 529/00-10C	Siberian ARI	257	41.5	35.1	+	+	-	+	+	+	+	-
97	Lutescens 36	Karabalyk AES	257	43.7	36.1	-	-	-	+	+	+	+	-
121	Lutescens 205/03-1	Kurganseeds	255	41.7	37.6	+	+	-	-	+	+	+	+

46	Lutescens - 1235	Karagandy ARI	255	43.3	36.4	-	-	+	+	+	+	+	+
145	Sigma	Siberian ARI	254	43.2	40.5	+	+	-	+	+	+	+	-
34	Stepnaya 1509/06	Aktobe AES	254	40.0	39.3	-	-	-	+	-	-	-	+
67	Lutescens 53/95-98-1	Pavlodar ARI	253	40.3	35.2	-	+	+	+	+	+	+	+
12	Lutescens 574	Altay ARI	252	40.8	36.9	-	+	-	+	+	+	+	+
3	Sibirskiy Aljans	Altay ARI	252	40.7	38.2	-	+	-	+	+	+	-	+
76	Lutescens 715-04	Kaz. Farming Inst.	251	42.5	40.9	-	+	-	+	+	-	+	-
22	Novosibirskaya 31	Novosibirsk ARI	251	40.8	31.2	-	-	-	-	-	+	-	-
104	Chelyaba yubileinaya	Chelyabinsk ARI	251	40.1	33.0	-	-	-	-	+	+	-	+
103	Ayna	Karabalyk AES	248	43.8	40.9	-	-	-	+	-	-	-	-
89	Fiton C-54	Fiton	245	42.5	41.5	-	-	-	+	-	+	-	-
99	Liniya-22 ЧС	Karabalyk AES	244	41.5	39.7	-	-	+	+	+	+	-	-
9	Lutescens 509	Altay ARI	244	40.0	40.6	-	-	-	+	+	+	+	-
72	Iljinskaya	Kaz. Farming Inst.	243	41.0	35.4	-	-	-	+	+	+	+	+
108	Pamyati Ryuba	Chelyabinsk ARI	243	42.3	39.1	-	-	-	-	+	+	+	-
149	Omskaya 35-2	Siberian ARI	242	40.6	38.6	-	-	-	-	+	-	+	-
13	Lutescens 697	Altay ARI	241	40.8	35.1	-	-	-	-	+	+	+	+
2	Altayskaya 530	Altay ARI	240	40.3	37.2	-	-	-	+	+	+	-	+
8	Lut.509	Altay ARI	238	40.0	39.2	-	-	-	+	-	+	+	-
105	Lutescens 23490	Chelyabinsk ARI	238	42.0	35.9	-	-	-	n.a.	+	+	-	+
56	Tselinnaya niva	Kaz. Grain Inst.	237	43.0	36.7	-	+	-	+	+	+	+	+
75	Lutescens 24	Kaz. Farming Inst.	236	40.0	39.7	-	+	-	+	+	+	+	-
100	Liniya-4-10-16	Karabalyk AES	235	43.3	33.3	-	-	-	+	-	+	-	+
53	Lutescens 1350	Karagandy ARI	230	40.3	37.3	-	+	-	+	+	+	+	+

83	Nadezhnda	Kaz. Farming Inst.	229	40.3	40.2	-	-	-	-	-	+	+	-
136	Chernyava 13	Omsk SAU	228	41.2	40.6	-	-	-	-	+	-	-	+
81	Kayir G-5454-91	Kaz. Farming Inst.	220	40.3	37.2	-	-	-	-	+	+	-	+
139	Tertsiya-1	Omsk SAU	199	42.0	33.0	-	-	-	-	+	+	-	-
4	Lutescens 844	Altay ARI	190	42.4	35.9	-	+	-	-	+	+	-	+
82	Kaz.stanskaya-3	Kaz. Farming Inst.	180	40.0	35.7	-	-	-	-	-	+	-	+
137	Sibakovskaya Yubilienaya	Omsk SAU	177	41.9	32.0	-	-	-	-	+	+	n.a.	-
Late heading group (44-48 days)													
21	Zauljbinka	East-Kaz. ARI	327	44.5	35.7	+	-	+	+	-	+	-	+
6	Toboljskaya-1	Altay ARI	302	44.8	38.4	-	+	-	+	+	+	+	+
7	Lutescens 1012	Altay ARI	301	44.5	37.9	-	+	-	+	+	+	-	+
69	Pavlodarskaya 11	Pavlodar ARI	298	44.3	34.6	-	-	-	-	+	+	+	-
40	LD-25	Saratov ARI	293	45.5	37.8	-	+	+	+	+	+	+	+
142	Lut. 3/04-21-11	Siberian ARI	291	44.5	39.7	+	+	-	-	+	+	+	+
141	Lutescens 141/03-2	Siberian ARI	291	45.1	40.5	+	-	-	+	-	+	+	+
120	Lutescens 360/96-6	Kurganseeds	290	44.4	37.2	+	+	-	+	+	+	-	+
134	OmGAU-100	Omsk SAU	285	44.5	36.8	+	+	-	-	+	+	+	+
39	L-654	Saratov ARI	284	46.2	33.7	-	n.a.	+	+	+	+	-	-
10	Lutescens 1003	Altay ARI	284	45.9	38.2	-	+	+	+	+	+	+	+
17	Velyutinum 15	East-Kaz. ARI	276	45.5	32.9	+	n.a.	+	+	+	+	+	+
44	Lutescens - 1212	Karagandy ARI	271	44.5	39.2	-	+	-	+	+	+	+	+
16	Lutescens 716	Altay ARI	270	44.7	34.0	-	+	-	+	+	+	+	+
150	Omskaya 41	Siberian ARI	269	46.0	35.6	+	n.a.	-	+	-	-	+	+
55	Lutescens - 1135	Karagandy ARI	267	45.6	34.2	+	n.a.	+	+	+	+	-	+
11	Erythrospermum 1119	Altay ARI	263	45.1	37.3	-	+	+	+	-	+	+	-

13<br>3	Lutescens 96-12	Omsk SAU	256	45.2	36.8	+	-	-	-	-	+	+	+
93	Fiton C 41 ЧС	Fiton	252	45.8	40.0	-	-	+	+	-	+	-	+
86	Fiton 43	Fiton	249	47.0	42.7	+	+	+	+	+	+	+	+
96	Lutescens C 19 ЧС	Karabalyk AES	239	47.4	31.1	+	n.a.	+	-	-	+	+	-
87	Fiton C 50 ЧС	Fiton	222	47.8	39.0	+	n.a.	+	-	-	+	+	-
